# Traumatic pneumopericardium following a suicide attempt by high fall in an adolescent girl

**DOI:** 10.1093/jscr/rjaf684

**Published:** 2025-09-02

**Authors:** Souha Qarouach, Hadjar Nassiri, Hidaya Zitan, Loubna Aqqaoui, Houda Oubejja, Fouad Ettayebi

**Affiliations:** Pediatric Surgical Emergency Department, Rabat Children's Hospital, Avenue Ibn Rochd, Rabat 10000, Rabat-Salé-Kenitra, Morocco; Faculty of Medicine and Pharmacy, Mohamed V University, Rabat, Avenue Mohammed Belarbi El Alaoui, Souissi, Rabat 10170, Rabat-Salé-Kenitra, Morocco; Pediatric Surgical Emergency Department, Rabat Children's Hospital, Avenue Ibn Rochd, Rabat 10000, Rabat-Salé-Kenitra, Morocco; Faculty of Medicine and Pharmacy, Mohamed V University, Rabat, Avenue Mohammed Belarbi El Alaoui, Souissi, Rabat 10170, Rabat-Salé-Kenitra, Morocco; Laboratory of Life and Health Sciences, Faculty of Medicine and Pharmacy of Tangier, Abdelmalek Essaadi University, Ziaten Road, PO Box 416, Tangier 90000, Tangier-Tetouan-Al Hoceima Region, Morocco; Pediatric Surgical Emergency Department, Rabat Children's Hospital, Avenue Ibn Rochd, Rabat 10000, Rabat-Salé-Kenitra, Morocco; Faculty of Medicine and Pharmacy, Mohamed V University, Rabat, Avenue Mohammed Belarbi El Alaoui, Souissi, Rabat 10170, Rabat-Salé-Kenitra, Morocco; Pediatric Surgical Emergency Department, Rabat Children's Hospital, Avenue Ibn Rochd, Rabat 10000, Rabat-Salé-Kenitra, Morocco; Faculty of Medicine and Pharmacy, Mohamed V University, Rabat, Avenue Mohammed Belarbi El Alaoui, Souissi, Rabat 10170, Rabat-Salé-Kenitra, Morocco; Pediatric Surgical Emergency Department, Rabat Children's Hospital, Avenue Ibn Rochd, Rabat 10000, Rabat-Salé-Kenitra, Morocco; Faculty of Medicine and Pharmacy, Mohamed V University, Rabat, Avenue Mohammed Belarbi El Alaoui, Souissi, Rabat 10170, Rabat-Salé-Kenitra, Morocco; Pediatric Surgical Emergency Department, Rabat Children's Hospital, Avenue Ibn Rochd, Rabat 10000, Rabat-Salé-Kenitra, Morocco; Faculty of Medicine and Pharmacy, Mohamed V University, Rabat, Avenue Mohammed Belarbi El Alaoui, Souissi, Rabat 10170, Rabat-Salé-Kenitra, Morocco

**Keywords:** pneumopericardium, high fall, adolescent, blunt chest trauma

## Abstract

Pneumopericardium is a rare complication of blunt chest trauma, particularly in pediatric patients. We report the case of a 14-year-old girl who sustained a fall from the fourth floor in a suicide attempt. She presented with hypotension and pelvic pain. Imaging revealed a pneumopericardium, a minimal pneumothorax, and vertebral and pelvic fractures. She underwent percutaneous decompression with favorable outcome. This case highlights the importance of considering pneumopericardium in pediatric trauma after high-energy falls.

## Introduction

Pneumopericardium, the accumulation of air in the pericardial sac, is a rare condition especially in children and adolescents. It is most often seen after blunt or penetrating chest trauma or as a complication of invasive mechanical ventilation [1]. Less commonly, it may also occur in the setting of infectious pericarditis [2]. When blunt trauma is involved, the leading causes are typically road traffic accidents or high falls [1].

We present the case of a teenage girl who sustained a fall from the fourth floor in a suicide attempt. She developed a pneumopericardium, multiple pelvic fractures and an L3 vertebral fracture. She was admitted to the pediatric emergency department, where she underwent percutaneous decompression with a favorable recovery.

## Case report

A 14-year-old female patient with no significant medical history was admitted to the pediatric emergency department 1 h after falling from the fourth floor in a context of a suicide attempt. Upon arrival, she was somnolent with a Glasgow Coma Scale score of 13, blood pressure of 80/50 mmHg, heart rate of 120 beats per minute, generalized pallor, and tachypnea at 25 breaths per minute. Clinical examination revealed no thoracic deformity or subcutaneous emphysema. The abdomen was soft and non-tender. Musculoskeletal assessment revealed pelvic pain with an inability to mobilize both lower limbs, although sensitivity was preserved.

**Figure 1:**
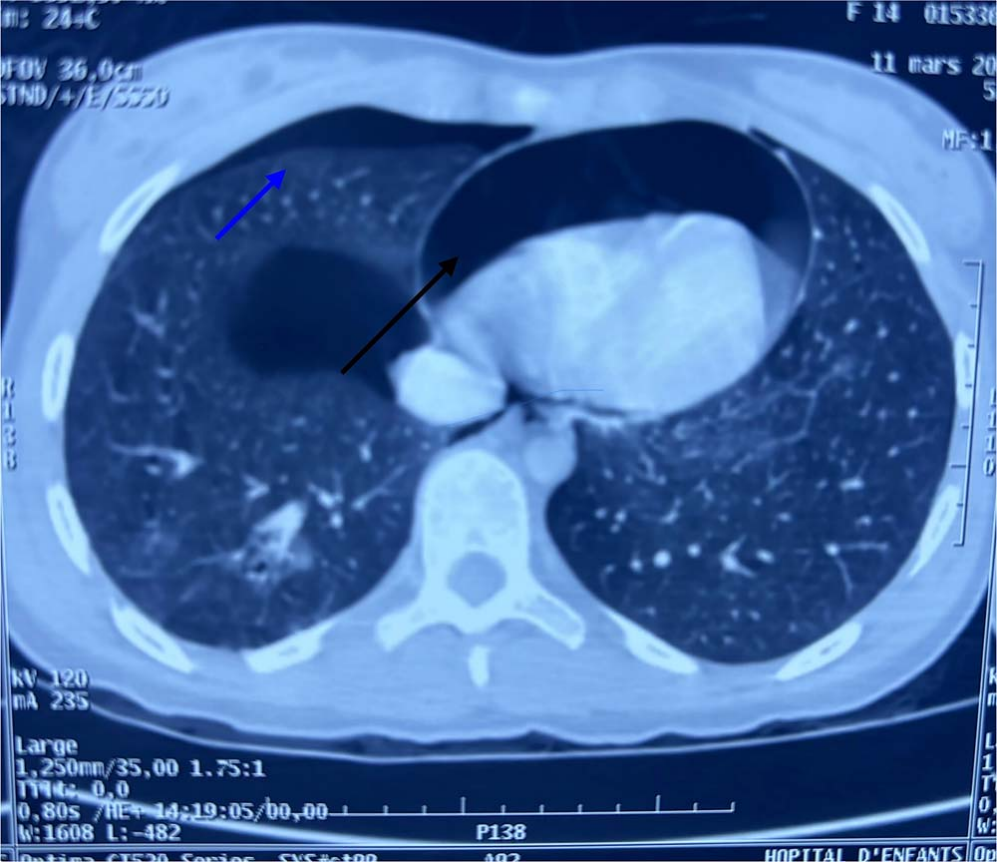
Axial chest CT scan showing pneumopericardium (lower arrow) and pneumothorax (upper arrow).

**Figure 2:**
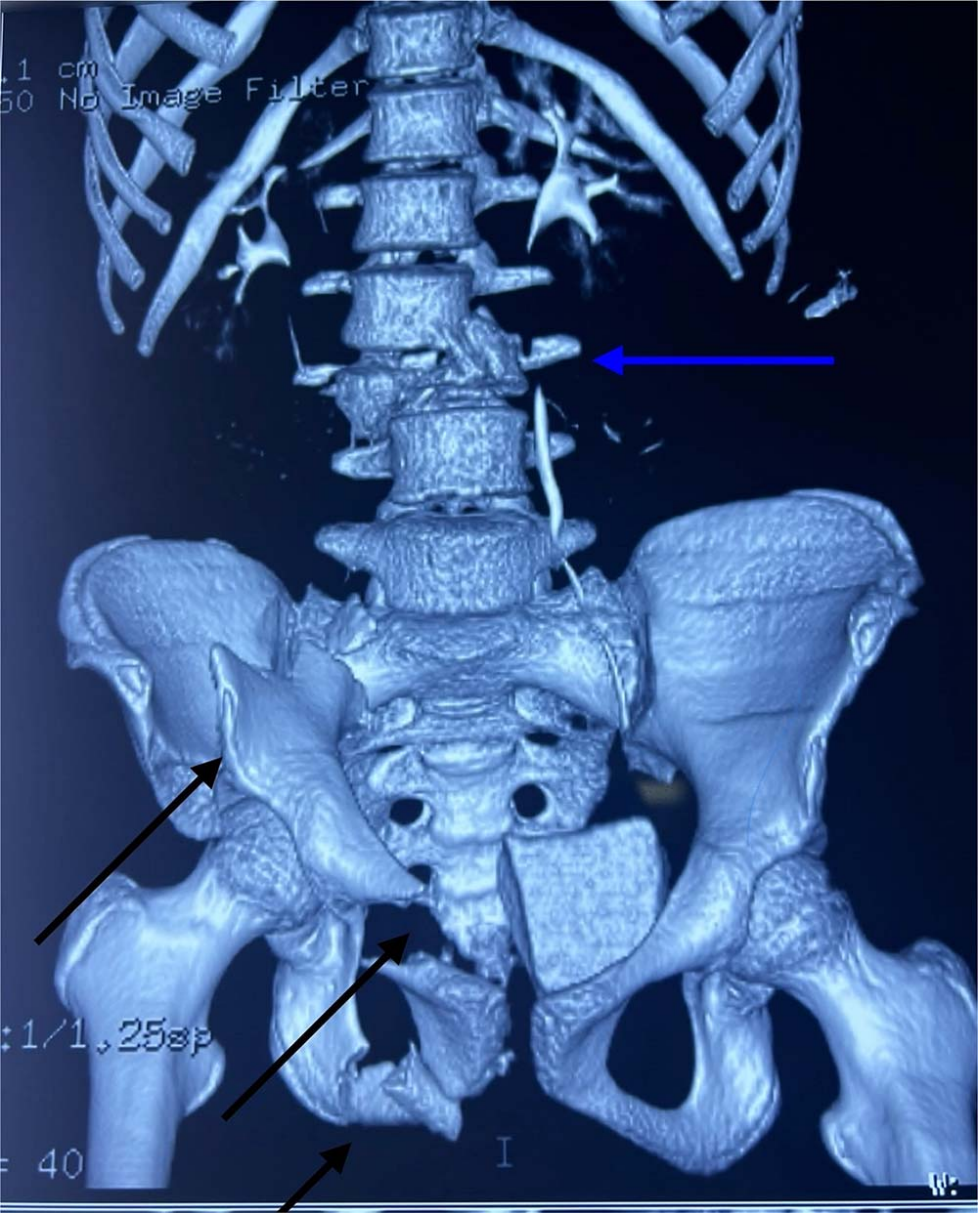
3D CT reconstruction showing L3 vertebral fracture (upper arrow) and multiple pelvic fractures (lower arrows).

After hemodynamic stabilization, an emergency full-body computed tomography (CT) scan was performed, revealing a minimal pneumothorax, a significant pneumopericardium (Fig. 1), multiple pelvic fractures, and a vertebral fracture at the L3 level (Fig. 2). Blood tests showed a hemoglobin level of 8 g/dL, prompting transfusion of two units of packed red blood cells. Following the onset of chest pain, a percutaneous decompression of the pneumopericardium was performed, resulting in rapid clinical improvement. The patient was then admitted to the intensive care unit for close monitoring. She subsequently underwent surgical osteosynthesis from L2 to L4.

## Discussion

Pneumopericardium, defined as the presence of air within the pericardial sac, is a rare condition first described by Bricheteau in 1844 [3]. Its known causes include penetrating or blunt chest trauma, mechanical ventilation, and a variety of other factors such as fistulas, iatrogenic injuries, or certain respiratory diseases [1]. What makes the present case particularly remarkable is its occurrence in an adolescent following a high-altitude fall—an event scarcely reported in pediatric literature.

Two main pathophysiological mechanisms are recognized. The first involves the formation of a fistula between an air-containing cavity (such as the lung, bronchial tree, or digestive tract) and the pericardium. This was the likely scenario in the present case, typically presenting with a pneumothorax and a pericardial tear, which allows air to enter the pericardial sac directly. The second mechanism, known as the Macklin effect, occurs when a sudden rise in intrathoracic pressure causes alveolar rupture. This allows air to escape into the pulmonary interstitium, track through the mediastinum, and possibly enter the pericardium if the visceral pleura remains intact [4, 5]. Infectious causes are exceedingly rare.

In their literature review, Capizzi *et al*. identified 32 cases over a 65-year period (1931–1995), most of which followed falls or motor vehicle accidents [1]. High-altitude falls whether accidental or intentional [6, 7] are associated with a high mortality rate, closely linked to the height of the fall [8], and are frequently accompanied by thoracic injuries [9, 10].

Although most of the available data concern adult patients, pediatric cases remain exceptionally rare. To date, only a few cases in children have been reported in the literature. These include an 11-year-old boy who developed pneumopericardium associated with pneumomediastinum following blunt chest trauma during a rugby match [11], as well as two additional cases reported by Shorr *et al*. in a series on tension pneumopericardium, in which two of the patients were adolescent [12]. These are observations highlight the importance of reporting new cases to improve clinical recognition and management of traumatic pneumopericardium in children.

Although pneumopericardium is often benign, it can evolve into a life-threatening tension pneumopericardium. Rapid accumulation of air may lead to cardiac tamponade. Adcock *et al*. showed that hemodynamic changes could occur with as little as 150 mL of air [13], and the speed at which air accumulates also influences clinical tolerance [14]. In Cummings’ study, 37% of pneumopericardium cases progressed to tamponade, with a mortality rate of 56% [15]. Capizzi’s series reported signs of tension in 12 of 32 patients, especially in those who were intubated or had an associated pneumothorax [1].

Typical symptoms of tension pneumopericardium include retrosternal chest pain (reported in 80%–90% of cases), dyspnea (50%), and Hamman’s sign a crepitus heard in sync with the heartbeat [16–18]. These symptoms can worsen with coughing, deep inspiration, swallowing, or lying flat, due to the accumulation of air in the mediastinum or pericardium [19].

Imaging plays a crucial role in diagnosis. Chest X-rays often show a pericardial air band (the ‘halo sign’) and thickened pericardial contours. However, the coexistence of pneumopericardium with pneumomediastinum or pneumothorax can complicate diagnosis. As Cimmino pointed out, many cases of pneumopericardium were actually misdiagnosed as pneumomediastinum [20]. When uncertainty remains, a chest CT scan provides a clearer view of the air leak’s origin [21].

A simple, asymptomatic pneumopericardium may resolve spontaneously with close monitoring. However, if tension pneumopericardium is suspected or confirmed, urgent decompression is required [22, 23]. In our case, percutaneous drainage effectively relieved the pneumopericardium.

This case underscores the need for high suspicion of pneumopericardium in pediatric trauma, especially after high-energy mechanisms such as falls.

## Conclusion

Pneumopericardium is an uncommon but potentially life-threatening complication of blunt chest trauma, especially in children and adolescents. Prompt recognition and timely treatment are essential to avoid progression to tension pneumopericardium. This case serves as a reminder to keep pneumopericardium in mind when evaluating young patients with chest injuries following a significant fall.

Further case documentation will aid in refining diagnostic and management algorithms for pediatric pneumopericardium.
